# Peripheral Blood Mononuclear Cells as a Laboratory to Study Dementia in the Elderly

**DOI:** 10.1155/2014/169203

**Published:** 2014-04-30

**Authors:** Beatrice Arosio, Claudio D'Addario, Cristina Gussago, Martina Casati, Enzo Tedone, Evelyn Ferri, Paola Nicolini, Paolo D. Rossi, Mauro Maccarrone, Daniela Mari

**Affiliations:** ^1^Geriatric Unit, Fondazione Ca' Granda, IRCCS Ospedale Maggiore Policlinico, Via Pace 9, 20122 Milan, Italy; ^2^Geriatric Unit, Department of Medical Sciences and Community Health, University of Milan, Via Pace 9, 20122 Milan, Italy; ^3^Faculty of Bioscience and Technology for Food, Agriculture and Environment University of Teramo, Piazza Aldo Moro 45, 64100 Teramo, Italy; ^4^Department of Clinical Neuroscience, Karolinska Institutet CMM L8:01, 17176 Stockholm, Sweden; ^5^European Center for Brain Research, Santa Lucia Foundation, IRCCS, Via del Fosso di Fiorano 64, 00143 Rome, Italy; ^6^Center of Integrated Research, Campus Bio-Medico University of Rome, Via Alvaro del Portillo 21, 00128 Rome, Italy

## Abstract

The steady and dramatic increase in the incidence of Alzheimer's disease (AD) and the lack of effective treatments have stimulated the search for strategies to prevent or delay its onset and/or progression. Since the diagnosis of dementia requires a number of established features that are present when the disease is fully developed, but not always in the early stages, the need for a biological marker has proven to be urgent, in terms of both diagnosis and monitoring of AD. AD has been shown to affect peripheral blood mononuclear cells (PBMCs) that are a critical component of the immune system which provide defence against infection. Although studies are continuously supplying additional data that emphasize the central role of inflammation in AD, PBMCs have not been sufficiently investigated in this context. Delineating biochemical alterations in AD blood constituents may prove valuable in identifying accessible footprints that reflect degenerative processes within the Central Nervous System (CNS). In this review, we address the role of biomarkers in AD with a focus on the notion that PBMCs may serve as a peripheral laboratory to find molecular signatures that could aid in differential diagnosis with other forms of dementia and in monitoring of disease progression.

## 1. Introduction

The prevalence of dementia has increased globally, most noticeably in the ageing populations of the developed world. Alzheimer's disease (AD) is the most common type of dementia (60% of cases). Individuals affected by AD are 5.4 million in the United States and more than 33.9 million worldwide [1]. Moreover, AD prevalence is estimated to triple over the next 40 years and this will place a heavy burden on society and its health-care systems in terms of both economic costs and human impacts. The steady and dramatic increase in the incidence of AD and the lack of effective treatments have stimulated the search for strategies to prevent or delay its onset and/or progression.

There is general agreement that the epidemiological impact of dementia can be reduced by detecting and treating classical vascular risk factors since different studies provide evidence in favour of a coexistence of vascular and degenerative components in its pathogenesis [2].

In western countries vascular dementia (VD) is the second most common cause of dementia after AD among the elderly. A meta-analysis of the European studies on the incidence of dementia showed that VD constitutes 17.6% of all dementias [3]. In Europe and North America, AD is more common than VD in a 2 : 1 ratio; in contrast, in Japan and China VD accounts for almost 50% of all dementias. Also, the possibility of concomitant AD often confounds the relationship between cerebrovascular disease and VD.

AD is characterized by neurofibrillary tangles (NFT) and extracellular amyloid deposits. The former are composed of intraneuronal aggregates of hyperphosphorylated tau proteins and the latter are made of amyloid-beta (A*β*) peptides stemming from the sequential cleavage of a transmembrane precursor named amyloid precursor protein (APP).

Vascular pathology, namely arteriosclerosis, endothelial proliferation, and neovascularization, have been often found to be associated with NTF and amyloid plaques [4].

A number of autopsy studies have confirmed that among cases of dementia, AD-related pathology was associated with vascular lesions in nearly one-third of cases [5]. In addition, many epidemiological reports have demonstrated that the presence of vascular factors increases the risk of developing AD.

However, it is still a matter of controversy whether neurodegenerative AD-like disease and cerebrovascular lesions are coexisting but unrelated pathologies or whether they represent different results of synergistic pathogenic mechanisms.

It is hypothesized that an alteration of the neurovascular unit, which is the functional unit encompassing vascular cells, astrocytes, and perivascular neurons, is an early event in the pathogenesis of AD [6]. Dysfunction of the neurovascular unit results in impaired blood brain barrier (BBB) functions, dysregulation of cerebral blood flow, and impairment of A*β* clearance leading to an increase of oligomers and soluble A*β* forms [7]. Vascular oxidative stress and inflammation underlie many of these deleterious effects and are potential therapeutic targets even if, at present, there is no cure for AD and only a few medications aimed at slowing down memory deficits and clinical symptoms are available, with limited benefits.

Consequently, there is a pressing need for the identification of biomarkers that will aid in the differential diagnosis between AD and other forms of dementia and that will allow the detection of AD at early stages. Within the scenario of dementia, biomarker research may thus play an important role in paving the way towards novel diagnostic or therapeutic strategies.

## 2. Biomarkers

A biomarker is a characteristic that can be objectively measured and evaluated as an indicator of normal biological processes, pathogenic processes, or pharmacological responses to a therapeutic intervention [8, 9]. Many tests commonly used in clinical practice are biomarkers; biochemical tests provide soluble biomarkers, whereas physiological assessment and imaging measures provide anatomical and functional biomarkers. The majority have been identified on the basis of biological insight or underlying physiology. With increasing knowledge and practical experience, many of these tests have evolved into measurable end points in clinical research, applied as indicators of change, be it for the better or for the worse [10].

Biomarkers have also gained an important role in the field of clinical management and have established a close link with bedside medicine, by providing metrics of quality in medical care alongside meaningful costing. With effective translation into many clinical guidelines, biomarkers can facilitate the delivery of evidence-based medical care [11].

The evaluation of biomarkers may aid in the identification of diseases and may also allow correlations to be made with the progression or the susceptibility to a disease or a given treatment.

Yet, single biomarkers are unlikely to capture the complex process of human pathophysiology. Thus research may need to be geared towards sets of biomarkers, reflecting different, but intercalated, processes, which may enable a better assessment of disease states.

Biomarkers can be measured, for instance, in saliva, sweat, breath, blood/serum, urine, and cerebrospinal fluid (CSF). The fact that the collection of these biological fluids is significantly less invasive than biopsies is an important practical issue when studying neurodegenerative disorders like AD [12].

It has been reported that the sensitivity (definitely positive/(definitely positive + false negative)) of an “ideal” biomarker to detect AD should be at least 85%. Similarly, the specificity (definitely negative/(definitely negative + false positive)) in differentiating AD patients from controls and from patients with other forms of dementia should be at least 75% [10].

### 2.1. Biomarkers and Alzheimer

Despite the enormous advances in modern medicine, the diagnosis of AD remains largely clinical, based on patient history/examination, neuropsychological testing, and imaging techniques. Unfortunately the clinical diagnosis of AD suffers from limitations in that it only allows us to speak of probable or possible AD [13] with a 93% sensitivity and a 55% specificity. Furthermore, the diagnosis becomes far more difficult in the case of early or unusual presentations of the disease.

With the expansion of current knowledge on AD and the increasing availability of technical tools, there is an emerging need for the development of accurate biochemical and imaging tests that support the diagnosis [14, 15]. In this context the diagnostic criteria for AD proposed in 2007 [14] highlight the usefulness of genetic studies since they would enable a definite diagnosis to be made based on the demonstration of mutations in any of the three genes responsible for autosomal dominant disease: the gene for APP on chromosome 21, for presenilin 1 (on chromosome 14), and for presenilin 2 (on chromosome 1).

As to the more prevalent sporadic cases of AD, the need for a biological marker has proven to be urgent, for both the diagnosis and monitoring of the disease [16, 17].

Indeed, an ideal biomarker for AD would assist in the identification of preclinical disease, early disease diagnosis, staging of disease progression, and response to treatment [18]. Early diagnosis and identification of preclinical AD are particularly important issues considering the development of underlying neuropathology in those yet to display clinical symptoms. In particular, Mild Cognitive Impairment (MCI) is a well-described prodromal state of cognitive decline preceding dementia, with an accelerated conversion to AD estimated at 10–12% per year [19].

Over the past decade, biomarker discovery has become a rapidly advancing area of AD research.

With the development of structural, functional, and molecular techniques, neuroimaging is increasingly being employed as a diagnostic and prognostic tool in AD. Quantitative magnetic resonance imaging (MRI) is used to assess neurodegenerative changes in AD, which include reducing whole brain volume and cortical thickness associated with ventricular enlargement [20]. Early degeneration is also apparent in the hippocampus, entorhinal cortex, and medial temporal lobe of AD patients relative to controls [21]. In fact, MRI-determined hippocampal atrophy is currently the most established structural biomarker for AD and has been shown to predict the conversion from MCI to AD in about 80% of cases [22]. Additional neuroimaging techniques include functional MRI (fMRI), positron emission tomography (PET), and single-photon emission computed tomography (SPECT) which reveal abnormalities in brain synaptic activity, metabolism, and perfusion, respectively. Recent advances include the development of a number of amyloid-binding compounds, the most extensively studied being 11C-PIB (Pittsburgh Compound B, PIB). Several PET studies have detected an increased uptake of PIB in AD patients, which was found to correlate with the extent of cerebral atrophy and memory impairment [23]. Notably, longitudinal studies suggest that PIB imaging is able to predict the progression from normal cognition and MCI to symptomatic AD [24].

In view of the close relationship of the CSF with the brain and spinal cord, it is believed that the composition of this fluid may reflect biochemical changes in the CNS and thus provide information on the pathological changes occurring in neurodegenerative disorders [18]. Multiple studies have examined CSF for potential AD biomarkers. It is generally recognized that AD subjects compared to age-matched controls exhibit decreased CSF levels of soluble A*β*42 and increased CSF levels of total tau and phosphotau [25]. Importantly, diagnostic accuracy is improved by using the tau/A*β*42 ratio instead of either single biomarker, and this is reflected in an increase in sensitivity and specificity to 86% and 97%, respectively [26]. Moreover, this combination also appears to predict the subsequent development of AD in both cognitively normal and MCI patients [27, 28]. These findings have thus established CSF A*β*42 and tau as the most sensitive and specific diagnostic and predictive biomarkers for AD.

It should however be remarked that although neuroimaging and CSF biomarkers seem to be the most promising, they also carry some limitations. They are generally expensive to perform routinely and lumbar puncture is invasive and often unpleasant. Moreover a large variability exists in the literature as to CSF biomarker diagnostic accuracies and cut-offs, hampering or delaying their everyday application in the clinical setting [29, 30] and their potential use as indicators of prodromal AD.

Also, it is worth noting that the process of biomarker discovery involves many critical steps including study design, sample preparation, protein and peptide separation and identification, and bioinformatics and data integration issues that must be carefully controlled before achieving independent confirmation and validation.

Lastly, patient age is an important confounding factor in these biomarker studies and could explain some of the variability in published diagnostic accuracies and cut-offs [30]. Indeed a consistent number of subjects affected by Lewy Body Dementia (LBD), Frontotemporal Lobar Degeneration (FTLD), VD, and Corticobasal Degeneration (CBD) display an AD-like CSF biomarker profile [31].

## 3. Inflammation and Alzheimer

In the human brain several cell types are responsible for initiating and amplifying a specific inflammatory response. In AD signs of an inflammatory activation of microglia and astroglia are present both inside and outside amyloid deposits. Cell cultures and animal models suggest an interactive relationship between inflammatory response activation, reduced neuronal functioning, and amyloid deposition. Furthermore cells associated with extracellular plaques within AD brains can produce a variety of cytokines, chemokines, and other related proteins that influence plaque and tangle formation [32].

There is strong evidence that inflammation exacerbates neuronal loss [33, 34]. In fact, local inflammatory processes can exert a direct neurotoxicity, interfere with A*β* expression and metabolism, and maintain a chronic intracerebral acute phase protein secretion, which in turn favours formation of A*β* oligomers [35].

On the other hand, microglial activation leads to an increased brain expression of major histocompatibility complex type II and an increased secretion of proinflammatory cytokines and chemokines such as interleukin-1 (IL-1), interleukin-6 (IL-6), tumour necrosis factor-*α* (TNF-*α*), and interleukin-8 (IL-8), as well as complement components and acute phase proteins [36].

A “cytokine cycle” has been proposed where [37] the anti-inflammatory cytokines (IL-4, IL-10, and IL-13) regulate A*β*-induced microglial/macrophage inflammatory responses and modify the microglial activity surrounding amyloid neuritic plaques [38]. Such cytokines can inhibit the induction of IL-1, TNF-*α*, and MCP-1 in differentiated human monocytes and, above all, IL-10 causes dose-dependent inhibition of the IL-6 secretion induced by A*β* in these cells and in murine microglia [37].

Accordingly, several reports make it appears that the risk of AD is substantially influenced by polymorphisms in the promoter region and other untranslated regions, of genes encoding inflammatory mediators. Alleles that favour an increased or decreased expression of inflammatory mediators are more frequent in patients with AD than in controls [39].

A*β* has also been shown to induce a phagocytic response in microglia, suggesting a neuroprotective defense mechanism [40]. This is, however, coupled to an increased release of signalling molecules and reactive oxygen and nitrogen species, which may further promote neuronal damage [41].

Despite these findings, clinical trials of nonsteroidal anti-inflammatory drugs (NSAIDs) in AD patients have been disappointing [42].

### 3.1. Peripheral Blood Mononuclear Cells

Nowadays it remains the need for a reliable, minimally invasive, and inexpensive biomarker for dementia, leading many to investigate peripheral blood. Blood collection is simple, inexpensive, and less invasive than lumbar puncture, allowing for repeated sampling. Approximately 500 mL of CSF is absorbed into the blood daily [43] and there is also evidence for blood-brain barrier (BBB) dysfunction in AD and other neurodegenerative disorders, which may enhance protein exchange between both fluids [44]. Consequently, the leakage of CNS metabolites into the peripheral system may reflect neurodegenerative disease status and could offer a suitable source of disease biomarkers.

AD also affects PBMCs that are defined as any blood cell with a round nucleus (i.e., lymphocytes, monocytes, or macrophages). These blood cells are a critical component of the immune system which provide defence against infection and respond to intruders. The lymphocyte population consists of CD4+ and CD8+ T cells, B cells and natural killer cells, CD14+ monocytes, basophils, neutrophils, eosinophils, and dendritic cells.

**Figure 1:**
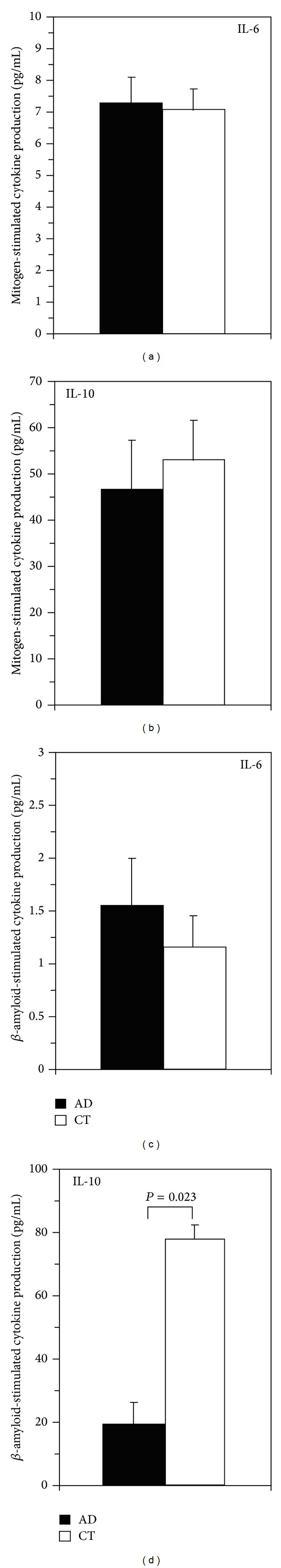
PBMCs of AD patients and age- and sex-matched controls (CT) were stimulated with a mitogen (LPS) and with a pool of three A*β* peptides (A*β* fragment 25–35; A*β* fragment 1–40; A*β* fragment 1–16). The production of IL-10 and IL-6 was measured by means of ELISA. There were no differences in mitogen-stimulated IL-6 and IL-10 production in AD and controls. In contrast, when A*β*-stimulated production of IL-6 and IL-10 was analysed, a marginally increased IL-6 production and a significantly decreased IL-10 generation were observed in AD patients compared to controls, suggesting an antigen-specific impairment in the production of these cytokines.

Although studies are continuously providing additional data that emphasize the central role of inflammation in AD, PBMCs have not been sufficiently investigated in this context. Indeed, only scant studies have used PBMCs to measure cytokine release, showing a significantly different production of these inflammatory components in AD and MCI subjects compared to controls [39, 45] (Figure 1) as well as a greater IL-1 and TNF-*α* production, associated with an increased risk of AD, in elderly controls [46].

Delineating biochemical alterations in AD blood constituents may enable the identification of accessible footprints that mirror degenerative processes within the CNS.

Moreover PBMCs could reflect inflammatory mechanisms in a more specific way compared with the serum/plasma, and PBMC-associated biomarkers could thus provide novel insight into the pathogenesis of AD.

In the following paragraphs we discuss the potential of PBMCs to serve as a peripheral laboratory to find molecular signatures in AD that could aid both in the differential diagnosis with other forms of dementia and in the monitoring of disease progression.

## 4. Peptidyl-prolyl **cis-/trans**-Isomerase Pin1 in PBMCs

The peptidyl-prolyl* cis-*/*trans-*isomerase Pin1 is a cytosolic protein that isomerizes the peptide bond of a phosphorylated serine or a phosphorylated threonine followed by a proline (pSer-/pThr-Pro). Pin1 catalyzes the* cis*-/*trans*-isomerization of its substrates, consequently potentiating the accessibility of the phosphate residue for further dephosphorylation by protein phosphatases such as the protein phosphatase PP2A. Alternatively, the binding of Pin1 to other highly phosphorylated substrates can repress their dephosphorylation by calcineurin. Therefore, through isomerization of pSer-/pThr-Pro, Pin1 regulates the function or degradation of a growing number of proteins including transcription factors and cytoskeletal, mitotic, or proapoptotic proteins [47].

Pin1 consists of 2 functional domains. The binding domain corresponds to the amino-terminal region consisting of a group IV WW domain (Trp-Trp domain) that specifically binds to pSer-/pThr-Pro motifs. The carboxyl-terminal region is the catalytic domain [48]. Pin1 substrate-binding and isomerase activity are regulated by phosphorylation. Indeed, 3 phosphorylation sites of Pin1 have been characterized. In particular, serine 16 is located in the WW domain and is phosphorylated by protein kinase A. Phosphorylation of Pin1 at serine 16 represses substrate recognition [49]. Pin1 has several additional putative phosphorylation sites (e.g., human Pin1 has 29 residues of serine or threonine and 3 tyrosines).

Phosphorylation of proteins is a key signalling mechanism in diverse of physiological and pathological processes. Pin1-catalysed conformational changes can have profound effects on phosphorylation signalling by regulating a spectrum of target activities. Interestingly, Pin1 deregulation is implicated in a number of conditions, notably ageing and age-related diseases, including cancer and AD. Pin1 is overexpressed in most human cancers; it activates numerous oncogenes or growth enhancers and also inactivates a large number of tumour suppressors or growth inhibitors. By contrast, ablation of Pin1 prevents cancer but eventually leads to premature ageing and neurodegeneration. Recent studies have demonstrated the reemergence within the brain of cell cycle proteins as patients progress from MCI into AD. Pin1 plays an important role in regulating the activity of key proteins, such as CDK5, GSK3-*β*, and PP2A, that are involved not only in the cell cycle but also in the phosphorylation state of Tau [50].

Indeed, Pin1 facilitates tau dephosphorylation [51] and regulates APP metabolism, thus providing additional support to the hypothesis that it has a neuroprotective function against AD [52–56].

It has been reported that Pin1 activity is repressed by oxidation in AD [52–58] and that Pin1 is localized to granular vesicles in AD and FTD but not to tau aggregates [55, 59–63].

It should be remarked that the expression and activity of Pin1 are tightly regulated at a transcriptional level and that a Pin1 gene polymorphism (−842G/C) has been found to be associated with reduced levels of Pin1 in blood cells and with an increased risk for AD in an Italian cohort [64].

Interestingly, a depletion of the soluble form of Pin1 has been described in neurons from AD subjects [57, 65] and differences in Pin 1 molecular and biochemical parameters have been reported in PBMCs from late-onset AD (LOAD) compared with control subjects [66].

In particular, in PBMCs from LOAD we observed a significant increase in Pin1 gene expression together with a significant decrease in gene promoter methylation [66].

This latter finding holds particular relevance, since so far little is known about epigenetic patterns in AD. Moreover, epigenetic mechanisms have already been proposed as markers of AD in PBMC-derived DNA [67] and it has been claimed that DNA methylation in peripheral cells could be taken as a model of epigenetic gene regulation in the brain [68].

**Figure 2:**
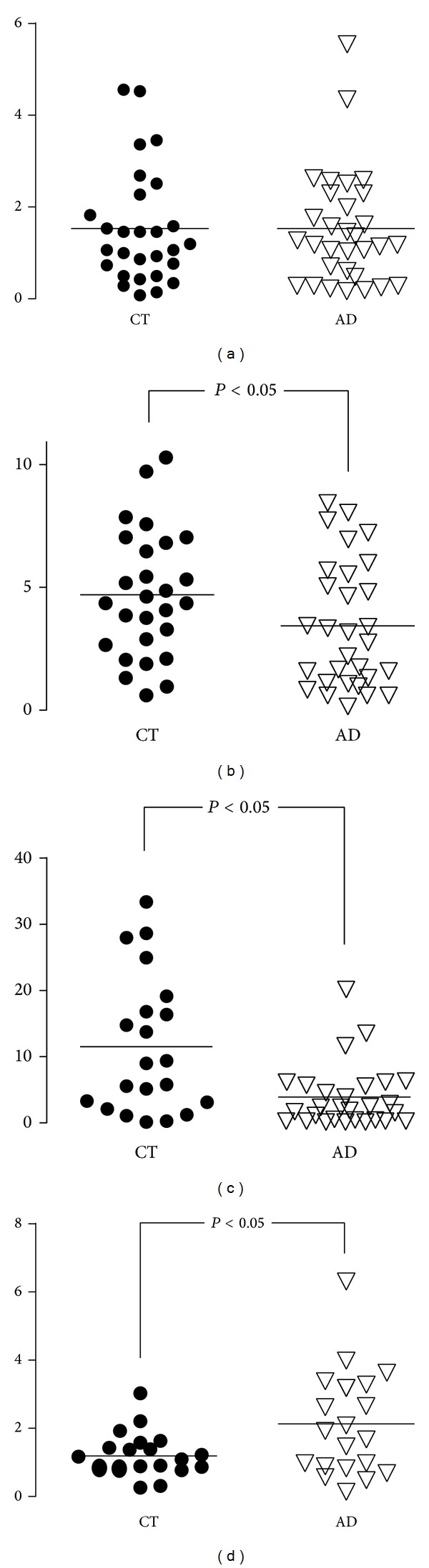
Scatter dot plots showing the distributions of molecular and biochemical parameters of PBMCs from controls (CT) and LOAD: activity (a), Ser^16^ phosphorylation (b), methylation (c), and gene expression (d). The lines across the boxes indicate median values.

We have also shown that in LOAD subjects Ser^16^ phosphorylation levels of Pin1 were lower than in controls (Figure 2).

Phosphorylation of Pin1 must therefore be a key factor in regulating its localization, function, and metabolism and tau seems to be involved in controlling the balance between the phosphorylation/dephosphorylation of Pin1 in brain cellular lysate [69].

Moreover, Wang et al. [70] suggested that reduced Pin1 activity in the frontal cortex of patients with MCI contributes to the initial accumulation of hyperphosphorylated tau and is then followed, in a more advanced stage of the disease, by a compensatory upregulation of the Pin1 gene that counteracts A*β* plaque formation.

In particular, with regard to our finding of lower Ser^16^ phosphorylation levels of Pin1 in LOAD subjects relative to controls, different interpretations can be put forward: the presence in LOAD patients of rare gene variants of Pin1 that could influence its phosphorylation state [71] and the effects on Pin1 of a higher blood concentration of A*β*42 [72]. In keeping with the latter hypothesis, in rat hippocampal cells, treatment with A*β*42 oligomers has been shown to promote a transient Pin1 dephosphorylation on Ser^16^ associated with a decrease in phosphorylated TauThr^231^ [73]. Whatever the specific explanation is, the modifications of Pin1 observed in LOAD subjects make it reasonable to suppose that Pin1 is involved in AD [74] and that epigenetic mechanisms (i.e., Pin1 promoter methylation) play a role in the disease. Therefore, alterations in easily accessible peripheral cells may prove to be valuable biomarkers in the diagnosis and follow-up of AD and, potentially, also of some tauopathies.

## 5. Epigenetics

Literally meaning “above the genome” the epigenome comprises the heritable changes in gene expression that occur in the absence of changes to the DNA sequence itself. Epigenetic mechanisms include chromatin folding and attachment to the nuclear matrix, packaging of DNA around nucleosomes, covalent modifications of histone tails, and DNA methylation in the whole genome and/or in specific gene promoters [75].

DNA methylation, in particular, consists of the transfer of a methyl group to position 5 of the cytosine pyrimidine ring of a cytosine guanine dinucleotide (CpG), which ultimately blocks the binding of transcription factors causing chromatin compaction and gene silencing [76].

The influence of regulatory small RNAs and microRNAs on gene transcription is also increasingly recognized as a key mechanism of epigenetic gene regulation [77].

Indeed, microRNAs (miRNAs), small regulatory RNAs in cells, probably constitute one of the most investigated extracellular RNAs in body fluidsand the levels of certain miRNAs in the circulation correlate well with different pathological conditions (i.e., miR-499 and miR-1 are associated with cardiovascular conditions) [78–81].

Epigenetic mechanisms are important in cell growth and differentiation [82]. Epigenetic change can be stochastic [83] or internally orchestrated as part of ageing [84]. Longitudinal changes in global and gene-specific DNA methylation clusters within families suggest there is a genetic control to methylation status [85].

Epigenetics is destined to change across the lifespan. In fact a loss of global DNA methylation and promoter hypermethylation of several specific genes occurs during ageing.

In particular ageing-associated DNA hypermethylation occurs predominantly in genes involved in the development of anatomical structures, organs, and multicellular organisms and in the regulation of transcription.

This phenomenon may be considered a new aspect of the age remodeling process, a continuous adaptation of the body to the deteriorative changes occurring over time. However, it is not clear how relevant these epigenetic changes are in the context of functional changes in gene expression [86].

Inappropriate epigenetic changes are associated with many diseases including cancer [87], Rett syndrome [88], Beckwith-Wiedemann syndrome [89], and other imprinting disorders.

Environmental signals can trigger epigenetic responses and may be an important mechanism by which environmental exposures are associated with disease [90]. Furthermore, epigenetic mechanisms may play an important role in the developmental origins of adult health and disease by providing a mechanism underlying the latent effects of adverse fetal, infant, and childhood environments on late-life chronic disease [91–93].

### 5.1. Epigenetic Epidemiology and Alzheimer's Disease

Epigenetic epidemiology is the study of the effects of heritable epigenetic changes on the occurrence and distribution of diseases in populations [94]. This research includes both transgenerational and intraindividual cellular epigenetic inheritance systems. Epigenetic changes are associated not only with ageing [95, 96], but also with psychiatric outcomes [97, 98] and neurodegeneration [99].

Evidence for the role of epigenetics in AD pathogenesis can be found in human studies of various tissues, in animal models, and in cell cultures [100–102]. Global changes associated with AD have been observed in DNA methylation, miRNAs, and histone modifications.

Discordant data have been reported on specific epigenetic modifications of tau- and amyloid-processing genes. On the one hand an altered regulation was reported across multiple brain regions [103–105], and on the other hand no differences were seen in DNA methylation in regions associated with MAPT, PSEN1, and APP [103].

Human postmortem case-control studies have demonstrated global hypomethylation in the entorhinal cortex of AD subjects [106] and in the temporal neocortex of an AD monozygotic twin relative to the cognitively normal twin [107].

An AD case-control study in the postmortem human parietal lobe cortex has revealed a differential regulation of several miRNAs, including miR-204, miR-211, and miR-44691 [108].

Age-matched AD cases have been found to exhibit an increased neuronal global phosphorylation of histone 3 relative to controls, as determined by immunolabeling in the hippocampus, and such histone modification suggests mitotic activation [109].

In experiments where neuroblastoma cells were cultured under low folate and vitamin B12 conditions, PSEN1 and BACE1 were hypomethylated, mRNA expression of BACE1 and PSEN1 was significantly induced, and A*β* production was increased [110].

An additional study using human neuroblastoma cells and male rat brain tissue reports that APP mRNA expression is repressed by thyroid hormone (T3) sensitive histone modifications [111].

### 5.2. Epigenetics in PBMCs

The study of gene regulation in blood cells from living patients offers the possibility to go through the whole history of the disorder (including the response to pharmacological, metabolic, and environmental events) in a more comprehensive perspective, compared to postmortem studies which allow only pinpoint assessment.

It is important to note that PBMCs may also be a useful model of epigenetic gene regulation in the brain [68]. In fact, it has been shown that PBMCs share much of the nonsynaptic biochemical environment of neurons and contain the full complement of epigenetic enzymes found in most tissues, including neurons and peripheral nucleated cells [112, 113].

For instance, our group has investigated the role of DNA methylation in the PBMCS from LOAD subjects compared to controls and has demonstrated an altered Pin1 gene expression and promoter methylation [66], as detailed above, along with changes in fatty acid amide hydrolase (FAAH) and 5-lipoxygenases (5-LOX) genes (Faah EC 3.5.1.99 and Alox5 EC 1.13.11.34), proteins, and activity [114].

Also, by comparing DNA methylation of Faah and Alox5 promoters we found a direct correlation between these two genes [114, 115].

It has been shown that oxygenation of the FAAH substrates by lipoxygenase activity modulates recognition of these molecules by their protein targets [116], with potential implications for their biological activity [117].

These results might suggest that a parallel increase of FAAH and 5-LOX expression in AD patients could evoke a sustained inflammatory condition, thus reinforcing neurodegeneration [114, 115].

This finding in peripheral cells is in agreement with previous results in postmortem AD brains [118], where FAAH protein upregulation within plaques was suggested to lead to an increase in metabolites from endocannabinoid anandamide (AEA) degradation (such as arachidonic acid). Such metabolites could contribute to the inflammatory process occurring in AD.

Recently, there has been considerable interest in exploring the therapeutic potential of anti-inflammatory agents to prevent, treat, or slow down the progression of AD [119]. However, nonsteroidal anti-inflammatory drugs were found to be ineffective in AD patients with mild to moderate cognitive impairment [120], emphasizing the importance of an early diagnosis and therapy. Furthermore, pharmacological interventions based on chronic treatment with COX inhibitors, or treatment with anticytokine therapies, are not ideal for a long-term use, due to their gastrointestinal (COX1-selective inhibitors), cardiovascular (COX2-selective inhibitors), and immunosuppressive (anticytokine therapies) side effects [121].

Taken together, these lines of research converge towards the notion that novel anti-inflammatory targets may provide a safer strategy for the prevention and the treatment of AD. In such scenario PBMCs stand out as potential peripheral markers of disease within the CNS.

## 6. Adenosine A_****2A****_ Receptors in PBMCs

Nutritional alterations have been linked to the epigenetic modulation of some AD-related genes and seem to play a role in AD pathology. There is also evidence in favour of the epigenetic modulation of genes involved in the pathways activated by some dietary factors, both in ageing and disease, further supporting the involvement of epigenetic mechanisms in AD. A number of dietary elements have been reported to be either risk or protective factors for the development of AD. These include fat, fatty acids, antioxidants, fish, vitamins, alcohol, and, more recently, caffeine [122].

The neuroprotective effect of caffeine consumption on AD pathology is currently emerging from both basic and epidemiological studies [123].* In vitro *and animal studies have provided convincing data on caffeine's neuroprotective effects against and in the presence of AD pathology [124–126]. Human studies have begun to demonstrate the presence of a similar neuroprotective role in the ageing and demented population.

However, due to the conflicting results from some longitudinal studies, there is no consensus about the role of caffeine in the onset of AD [124–128].

Caffeine is one of the most consumed psychoactive drugs and acts mostly by blocking adenosine receptors [129]. The purine ribonucleoside adenosine (Ado) is a naturally occurring metabolite that is ubiquitously distributed throughout the body as a metabolic intermediary. Intra- and extracellular Ado levels rise in response to physiological stimuli and with metabolic/energetic perturbations, inflammatory challenges, and tissue injury [130, 131].

The physiological responses to Ado take place as a result of the binding and activation of different transmembrane receptors: the high-affinity A_1_ and A_2A_ (A_2A_) receptors, the low-affinity A_2B_ receptor, or the low-abundance A_3_ receptor [132].

These receptors are G-protein coupled receptors that regulate, in opposite directions, the second messenger cAMP; while A_1_ is inhibitory Gi*-*coupled, A_2A_ is excitatory Gs-coupled, thereby decreasing and increasing cAMP levels, respectively [133]. The activation of these receptors is also able to modulate Ca^2+^ channels and the phospholipase C pathway. Through these actions and by modulating both the release and the uptake of different neurotransmitters, the balance between the activation of adenosine A_1_ and A_2A_ receptors allows the fine tuning of synaptic transmission and plasticity in the hippocampus [134].

In particular A_2A_ is present in a wide variety of tissues, including the nervous system and the peripheral immune system, where they display different levels of expression: significant levels in neurons and peripheral cells (lymphocytes and neutrophils) and lower levels in glial cells [132]. The different levels of expression of A_2A_ in different tissues are consistent with the sophisticated, multifaceted neurochemical, and molecular effects of the Ado system. On the basis of* in vitro* [135, 136] and* in vivo* [137] studies, it has become clear that A_2A_, through complex mechanisms which are still poorly understood [138–141], plays a critical role in the modulation of inflammatory reactions, influencing functional outcome in a broad spectrum of pathologies including neurodegeneration [142, 143].

Moreover it has been demonstrated that A_2A_ is able to prevent A*β*-induced synaptotoxicity in animal models and cell cultures [144] and it has been shown to control NMDA currents and glutamate outflow in the hippocampus [145, 146].

Contrasting data have been reported so far on the beneficial/detrimental effects of A_2A_ on brain cells [147]. The blockade of A_2A_ alleviates the long-term burden of brain disorders such as ischaemia, epilepsy, Parkinson's disease, or AD [138, 145, 148, 149]. On the other hand, agonists of A_2A_ can protect the CNS against several insults, including ischemia and excitotoxins [143, 150].

In the periphery A_2A_ contributes to coronary endothelial dilatation in mice [151], can inhibit endothelial apoptosis [152], and preserves vascular reactivity following hemorrhagic shock in rats [153].

**Figure 3:**
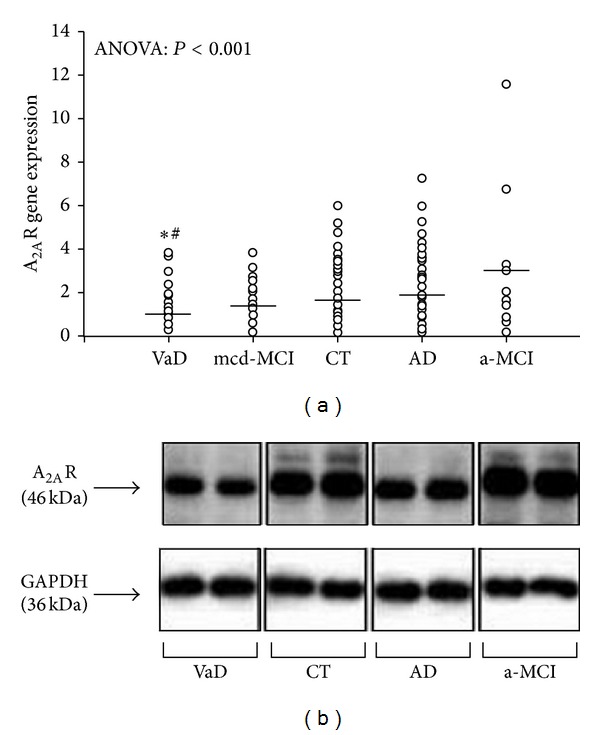
(a) Scatter plot of A_2A_ gene expression in PBMCs from VD, mcd-MCI, controls (CT), AD, and a-MCI subjects (the lines represent the mean value for each group). *P < 0.001 versus AD; ^#^ *P* < 0.05 versus a-MCI. (b) Representative picture of the western blot analysis of the A_2A_ densities in PBMCs extracts, running in duplicate, from one subject from the VD, CT, AD, and a-MCI groups, respectively.

We recently investigated A_2A_ gene expression and density in the PBMCs of patients with amnestic MCI (a-MCI), multiple cognitive domain MCI (mcd-MCI), outright AD, VD, and controls. We found that A_2A_ expression is upregulated in the peripheral cells of a-MCI but not AD subjects, supporting an involvement of the Ado system in the early stages of AD [154]. We also showed that A_2A_ expression is lower in the PBMCs of subjects with VD than AD, highlighting its possible relevance as a biomarker that may help differentiate two forms of dementia that are often closely associated (Figure 3).

Indeed, ROC analysis data showed that A_2A_ possesses a moderate degree of sensitivity and specificity for identifying VD patients from a heterogeneous group composed of VD and AD patients. The lower A_2A_ expression in VD compared to AD subjects seems to suggest a differential role of the Ado system in these dementias [155].

The methylation of the ADORA2A promoter gene, which codes for A_2A_, may explain its different expression in these pathological conditions as well as in the ageing process, as already mentioned [156].

Moreover, A_2A_ represents the main Ado receptor involved in inflammation and it is interesting to note that also other inflammatory biomarkers are differently expressed in VD and AD subjects, such as alpha1-globulin and alpha2-globulin in the serum [157] and C3a and C4a in the CSF [158].

On the other hand the decreased A_2A_ levels in VD could be a defence mechanism since it has been demonstrated that pharmacologic inactivation or genetic deletion of A_2A_R reduces neuronal injury after global and focal cerebral ischemia in many animal models [149, 159, 160]. From our results it can be concluded that A_2A_ may play an important but differential role in both types of dementia: its upregulation in the preclinical stages of AD could counterbalance the existing inflammatory state and its downregulation in VD could reflect the effects of A_2A_ on the brain vasculature [161]. It can therefore be suggested that A_2A_ could serve as a biomarker in the differential diagnosis between VD and AD.

## 7. Conclusions

Peripheral cells and in particular PBMCs seem to directly participate to neurodegenerative processes. They play critical roles in immune response, metabolism, and communication with other cells as already pointed out many years ago [162]. Moreover, PBMCs have been shown to share much of the nonsynaptic biochemical environment of neurons and contain the full complement of epigenetic enzymes and machinery, which are found in both neurons and peripheral nucleated cells, as in most other tissues.

The substantial evidence in favour of the notion that PBMCs provide a window into the CNS holds particular relevance in neurodegenerative disorders in which, unlike most other diseases, the affected tissue is not directly accessible to evaluation. On a final note, it should be mentioned that the value of biochemical dysfunctions in PBMCs as mirrors of CNS defects appears to extend well beyond dementia.For instance, FAAH and other elements of the endocannabinoid system show alterations in the blood that resemble those within the CNS in a broad spectrum of clinical conditions including Parkinson's and Huntington's disease, multiple sclerosis, schizophrenia, minor depression, and headache [163].

Nowadays we do not know if PBMCs biomarkers are better or worse than the CSF biomarkers. Our study is only a preliminary study, instead multiple studies have examined CSF to establish sensitivity and specificity of CSF biomarkers. Moreover, despite these many studies, a large variability exists in the literature as to CSF biomarker diagnostic accuracies and cut-offs. As biomarker discovery in PBMCs is an ongoing process and PBMCs biomarkers are still immature, we need further analysis to enlarge design population.

It will be also of relevance the possibility to utilize intracellular biomarkers in specific blood cell subpopulations. In fact the differences in between subjects could also be due to different composition of their PBMCs pools, even if separating PBMCs into subpopulations would not permit the cell-cell interactions required for activation of lymphocytes.

Finally, we assume that the combination of peripheral and CSF markers may be utilized to categorize patients since early stages of dementia and to understand mechanisms underlying dementia.
