# Corrigendum to “Peripheral Blood Mononuclear Cells as a Laboratory to Study Dementia in the Elderly”

**DOI:** 10.1155/2020/9175369

**Published:** 2020-09-07

**Authors:** Beatrice Arosio, Claudio D'Addario, Cristina Gussago, Martina Casati, Enzo Tedone, Evelyn Ferri, Paola Nicolini, Paolo D. Rossi, Mauro Maccarrone, Daniela Mari

**Affiliations:** ^1^Geriatric Unit, Fondazione Ca' Granda, IRCCS Ospedale Maggiore Policlinico, Via Pace 9, 20122 Milan, Italy; ^2^Geriatric Unit, Department of Medical Sciences and Community Health, University of Milan, Via Pace 9, 20122 Milan, Italy; ^3^Faculty of Bioscience and Technology for Food, Agriculture and Environment University of Teramo, Piazza Aldo Moro 45, 64100 Teramo, Italy; ^4^Department of Clinical Neuroscience, Karolinska Institutet CMM L8:01, 17176 Stockholm, Sweden; ^5^European Center for Brain Research, Santa Lucia Foundation, IRCCS, Via del Fosso di Fiorano 64, 00143 Rome, Italy; ^6^Center of Integrated Research, Campus Bio-Medico University of Rome, Via Alvaro del Portillo 21, 00128 Rome, Italy

The article titled “Peripheral Blood Mononuclear Cells as a Laboratory to Study Dementia in the Elderly” [1] was found to contain a substantial amount of material from previously published articles including the following sources:

- Manraj S. Bhamra and Nicholas J. Ashton, “Finding a pathological diagnosis for Alzheimer's disease: Are inflammatory molecules the answer?: Proteomics and 2DE”, Electrophoresis, 2012. 10.1002/elps.201200161. [2] (not cited)
- Kelly M. Bakulski, Laura S. Rozek, Dana C. Dolinoy, Henry L. Paulson and Howard Hu, “Alzheimer's Disease and Environmental Exposure to Lead: The Epidemiologic Evidence and Potential Role of Epigenetics”, Current Alzheimer Research (2012) 9: 563. 10.2174/156720512800617991. [3] (cited as reference [75])
- Cristina Gussago, “Il Recettore Adenosinico A2a Come Possibile Biomarcatore Nella Diagnosi Differenziale Delle Demenze Nell'anziano”, Università Degli Studi Di Milano. 10.13130%2Fgussago-cristina_phd2014-03-10. [4] (not cited)
- B. Arosio, A. Bulbarelli, S. Bastias Candia, E. Lonati, L. Mastronardi, P. Romualdi, S. Candeletti, C. Gussago, D. Galimberti, E. Scarpini, B. Dell Osso, C. Altamura, M. Maccarrone, L. Bergamaschini, C. D Addario, D. Mari. “Pin1 Contribution to Alzheimer's Disease: Transcriptional and Epigenetic Mechanisms in Patients with Late-Onset Alzheimer s Disease”, Neurodegenerative Diseases, 2012.10.1159/000333799. [5] (cited as reference [66])
- K. Ando, P. Dourlen, A. V. Sambo et al., “Tau pathology modulates Pin1 post-translational modifications and may be relevant as biomarker,” Neurobiology of Aging, vol. 34, no. 3, pp. 757–769, 2013. 10.1016/j.neurobiolaging.2012.08.004. [6] (cited as reference [69])

The authors apologise in particular for not acknowledging the work of Bhamra and Ashton (2012).
